# Postoperative follow-up of a case of atypical morning glory syndrome associated with persistent fetal vasculature

**DOI:** 10.1186/s12886-019-1154-6

**Published:** 2019-07-16

**Authors:** Heng Jiang, Youling Liang, Kejun Long, Jing Luo

**Affiliations:** 1 Department of Ophthalmology, The Second Xiangya Hospital, Central South University, 139 Middle Renmin Rd, Changsha, Hunan 410011 People’s Republic of China; 2 Hunan Clinical Research Center of Ophthalmic Disease, Changsha, Hunan 410011 People’s Republic of China

**Keywords:** Morning glory syndrome, Morning glory disc anomaly, Congenital optic disc dysplasia, Persistent hyperplastic primary vitreous, Persistent fetal vasculature, Retinal detachment, Vitrectomy

## Abstract

**Background:**

Morning glory syndrome is a relatively rare congenital optic disc anomaly that is often difficult to identify when associated with additional congenital ocular anomalies. This case report describes the diagnosis, treatment, and postoperative follow-up care of a young girl with morning glory syndrome accompanied by persistent fetal vasculature, retinal fold, and retinal detachment. Here, we also give a brief review of the relevant literature.

**Case presentation:**

A 5-year-old girl was referred to our clinic for a complaint of decreased vision for 6 months in the right eye. The best corrected visual acuity was hand motion in her right eye and 0.8 in her left eye. A fundus examination indicated vitreous opacities and scattered hemorrhages, as well as striped folds in the temporal retina of the affected eye. B-ultrasound and magnetic resonance imaging scans suggested that it could be a congenital dysplasia of the right eye. Pars plana vitrectomy was performed in the right eye. Morning glory syndrome associated with persistent fetal vasculature was confirmed in subsequent follow-up observation according to the fundus appearance, optical coherence tomography, and fundus fluorescein angiography imaging.

**Conclusions:**

The patient was diagnosed as morning glory syndrome associated with persistent fetal vasculature and retinal fold. The morning glory disc with the presence of retinal folds did not seem quite typical and that made the diagnosis difficult. This report stresses the importance of considering concurrent morning glory syndrome and persistent fetal vasculature. Vitrectomy may be beneficial in the management of the morning glory syndrome and persistent fetal vasculature if accompanied by retinal detachment in similar cases.

## Background

Morning glory syndrome (MGS) is a congenital optic disc anomaly. It was named by Peter Kindler who observed that the fundus resembled a blossoming morning glory [1]. The prevalence of MGS has been reported to be 2.6/100,000 [2]. The pathogenesis of this congenital defect is not fully understood [3]. MGS is possibly a congenital coloboma of the optic nerve head, but may also be related to glial tissue dysplasia at the center of the optic disc. Recent studies have hypothesized that primary mesenchymal abnormalities result in incomplete closure of the posterior scleral wall and aplasia of the lamina cribrosa, which lead to MGS [4]. The majority of patients are unilaterally affected [2, 4, 5], and have poor vision (hand motion to 0.02) due to impaired visual development. This may explain why strabismus is commonly seen in MGS [2, 6]. In MGS, esotropia has a higher incidence than exotropia [7], as esotropia is considered to be a congenital ocular maldevelopment that generally forms during fetal development to one and a half years old.

Persistent fetal vasculature (PFV), previously known as persistent hyperplastic primary vitreous, is among the most commonly seen congenital ocular developmental malformations, and is a result of the primary vitreous’ failure to regress [8]. PFV is often unilateral, sporadic, and characterized by white vascularized retrolental tissue, microphthalmia, severe intraocular hemorrhage, occasional retinal fold, and varying degrees of lenticular opacification [8].

Although cases of MGS or PFV are rare, diagnosing either disease is often not difficult. Here, we report a seldom seen case in which the patient presented an atypical fundus appearance of both diseases.

## Case report

**Fig. 1:**
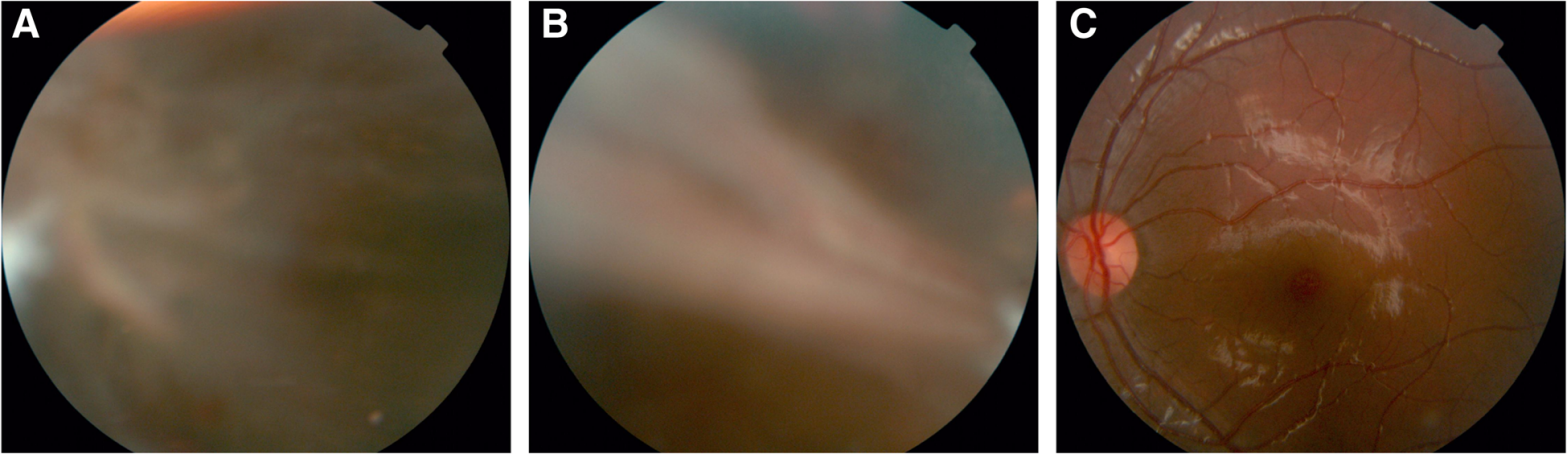
Binocular fundus photographs. **a** Grayish white retinal protuberance at the posterior pole of the RE. **b** Retinal folds appeared as white stripes in the temporal retina of the RE. **c** The fundus of the LE appeared normal with no vascular abnormalities and fibroplasia in the posterior pole or peripheral retina

A 5-year-old female patient visited our department on June 28, 2016 with a complaint of vision loss in the right eye for 6 months. The child was born at full-term by normal vaginal delivery and had no other relevant medical history, except for having contact with pets. There were no abnormal findings in the general physical examination. To be precise, her head size, facial appearance, or other significant indicators of the development of central nervous system were at the same level with normal children of the same age. Best corrected visual acuity (BCVA) was hand motion in the right eye (RE) and 0.8 in the left eye (LE). Intraocular pressure, measured by non-contact tonometer, was 15 mmHg and 14 mmHg in the RE and LE, respectively. A 20 prism dioptres esotropia was observed in the RE. Examination of the anterior segment was unremarkable and both lenses were transparent. During the initial fundus examination, we observed vitreous opacities and scattered hemorrhages distributed in the inferior peripheral portion. A grayish white retina at the posterior pole (Fig. 1a) and a few striped retinal folds at the temporal side could be vaguely seen (Fig. 1b). The LE did not present any distinct abnormalities (Fig. 1c).

**Fig. 2:**
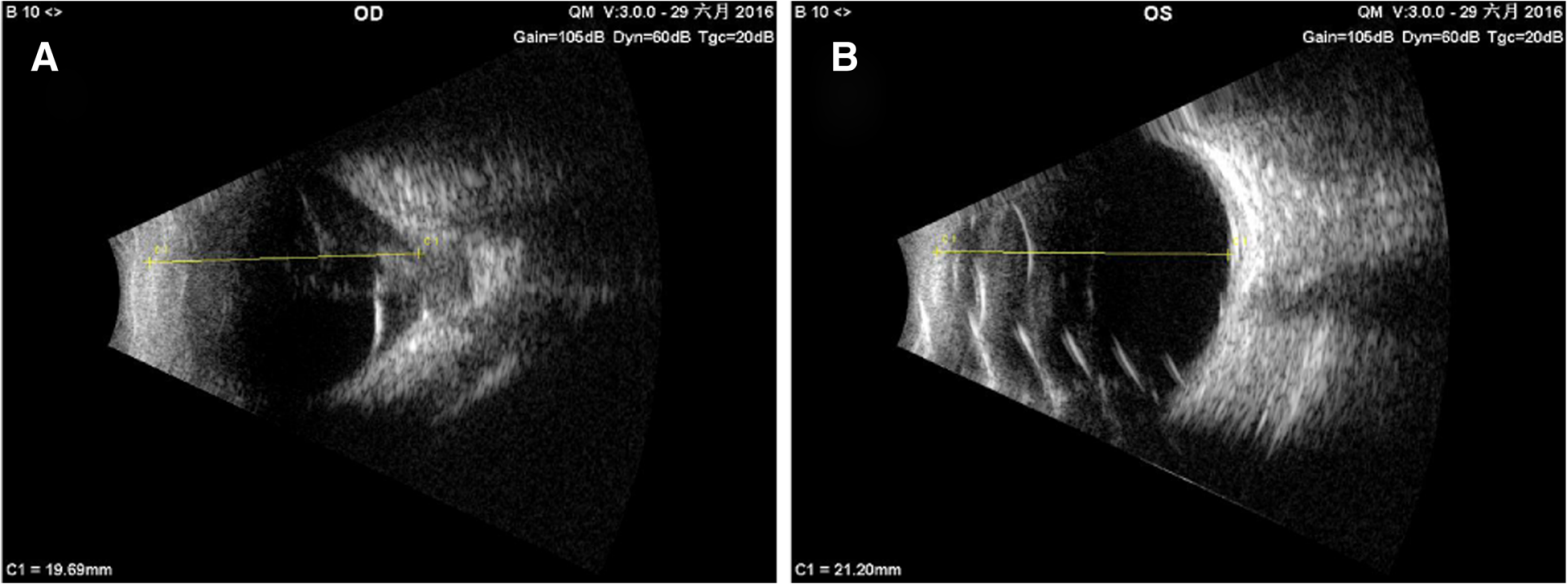
B-ultrasound scans of the RE (**a**) showed a depression in the optic disc filled with an unknown substance and adjacent hyperechoic peripapillary membrane elevation. The axial length measured by B-ultrasound was 19.69 mm in the RE (**a**) and 21.20 mm in the LE (**b**)

**Fig. 3:**
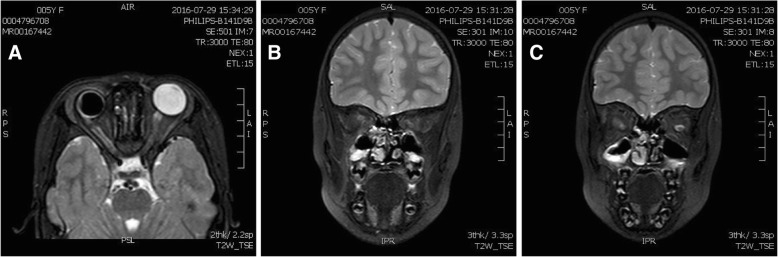
Cranial MRI in T2W_TSE. **a** A transverse section image showed mild microphthalmia and irregular thickening of the ocular wall of the RE. Dysplasia of the right optic nerve was also observed. The subarachnoid space surrounding the right optic nerve (optic nerve sheath) was prominently increased, however the signal intensity was normal. **b** and **c** A comparison of the intraorbital segment of the optic nerves indicated that the right optic nerve (**b**) was significantly smaller than the nearside (**c**)

In addition, a B-ultrasound scan showed a distinct depression at the optic disc that was filled with unidentified substance. Hyperechoic peripapillary membrane elevation was observed adjacent to the depression (Fig. 2a). Cranial MRI scans illustrated irregular thickening of the right ocular wall and mild microphthalmia of the RE (Fig. 3a). This corresponded to the axial length of both eyes (19.69 mm in the RE and 21.20 mm in the LE) measured by B-ultrasound scans (Fig. 2a, b). It appeared that the right optic nerve was thinner than normal, but no abnormal signal was detected in orbits or brain (Fig. 3a, b, c). These results suggested a possible dysplasia in the right eye and the right optic nerve.

To relief retinal detachment (RD) as well as to prevent the exacerbation of vision loss and eyeball atrophy, the patient underwent pars plana vitrectomy (PPV) on July 26, 2016. During the surgery, we found that a stalk arose from the optic disc and adhered to the peripheral retina and the vitreous proliferated along the Cloquet’s canal. Retinal folds arising from the optic disc and proliferative membranes were also found. Numerous fibrous membranes and the stalk were removed. Subsequently, we drained the subretinal fluid by a drainage retinotomy as no retinal break was seen. This was followed by endolaser photocoagulation surrounding the site of retinotomy, complete air-fluid exchange, and silicone oil tamponade. There was still some subretinal fluid and funds structures were unrecognizable as MGS at that time.

**Fig. 4:**
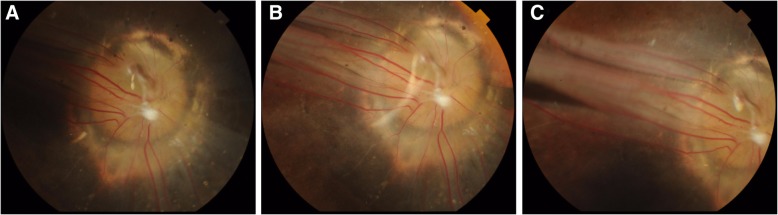
**a**, **b** and **c** Fundus photographs at the second postoperative follow-up presented a morning glory disc of the RE

The fundus became clearer as the subretinal fluid was absorbed over time. At the second visit, over a month after the surgery on September 13, 2016, the visual acuity of the RE improved from hand motion to counting fingers and could not be corrected with glasses. The right fundus resembled a morning glory disc with signs of PFV (Fig. 4a, b, c). The optic disc markedly enlarged to 4–5 PD in diameter. The deep depression in the optic disc was covered by a tuft of white glial tissue. Approximately 20 retinal vessels with differing widths radiated from the border of the white glial tissue into the peripheral retina. Arteries were difficult to differentiate from veins. Surrounding the optic disc was a wide circular yellowish-white to grayish-black eminence with pigment granules. Outside the eminence was a concentric retinochoroid atrophy ring extending into the macula. Striped retinal folds arose from the posterior pole and continued into temporal peripheral retina.

**Fig. 5:**
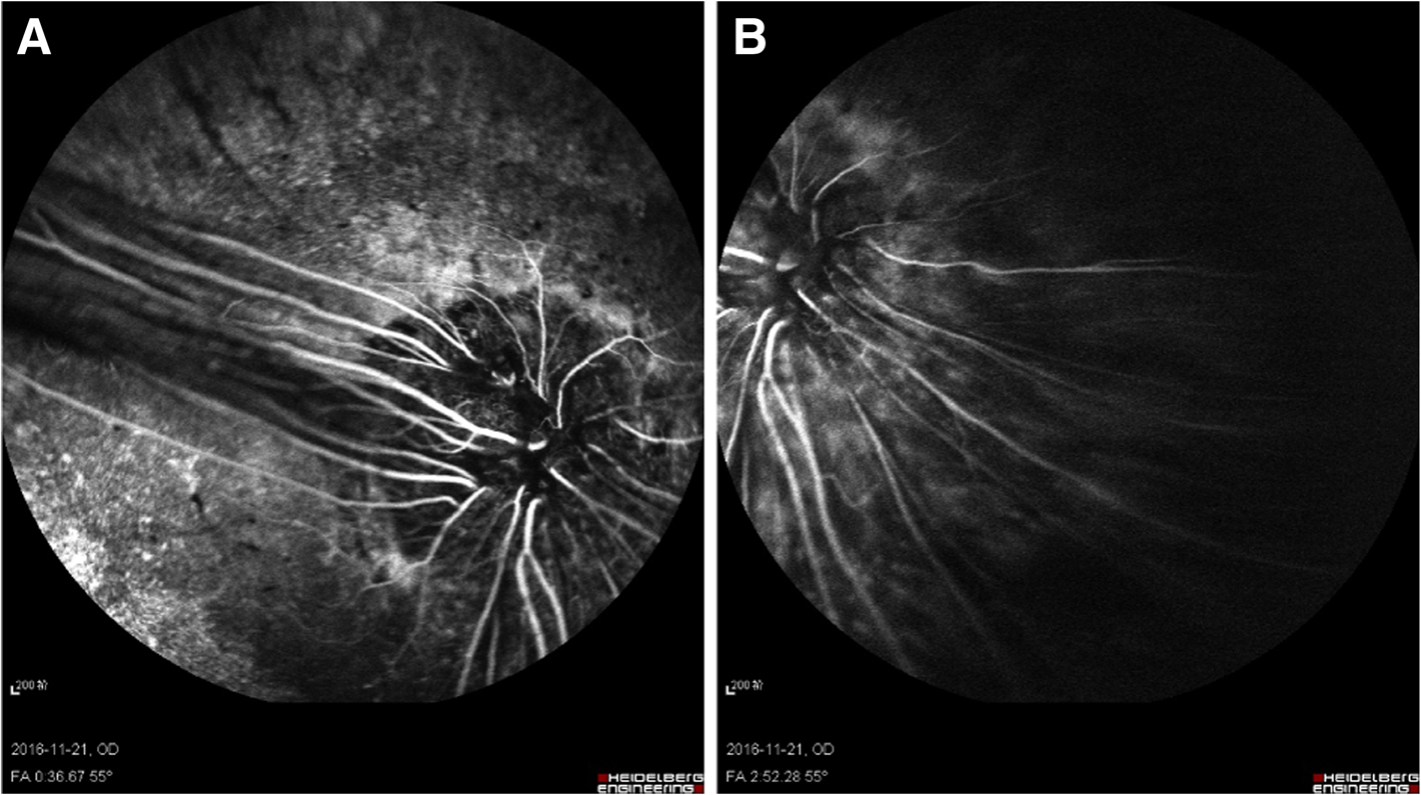
FFA of the RE. **a** and **b** The enlarged optic disc with vessels emanated from superotemporal and inferonasal margin of the disc

**Fig. 6:**
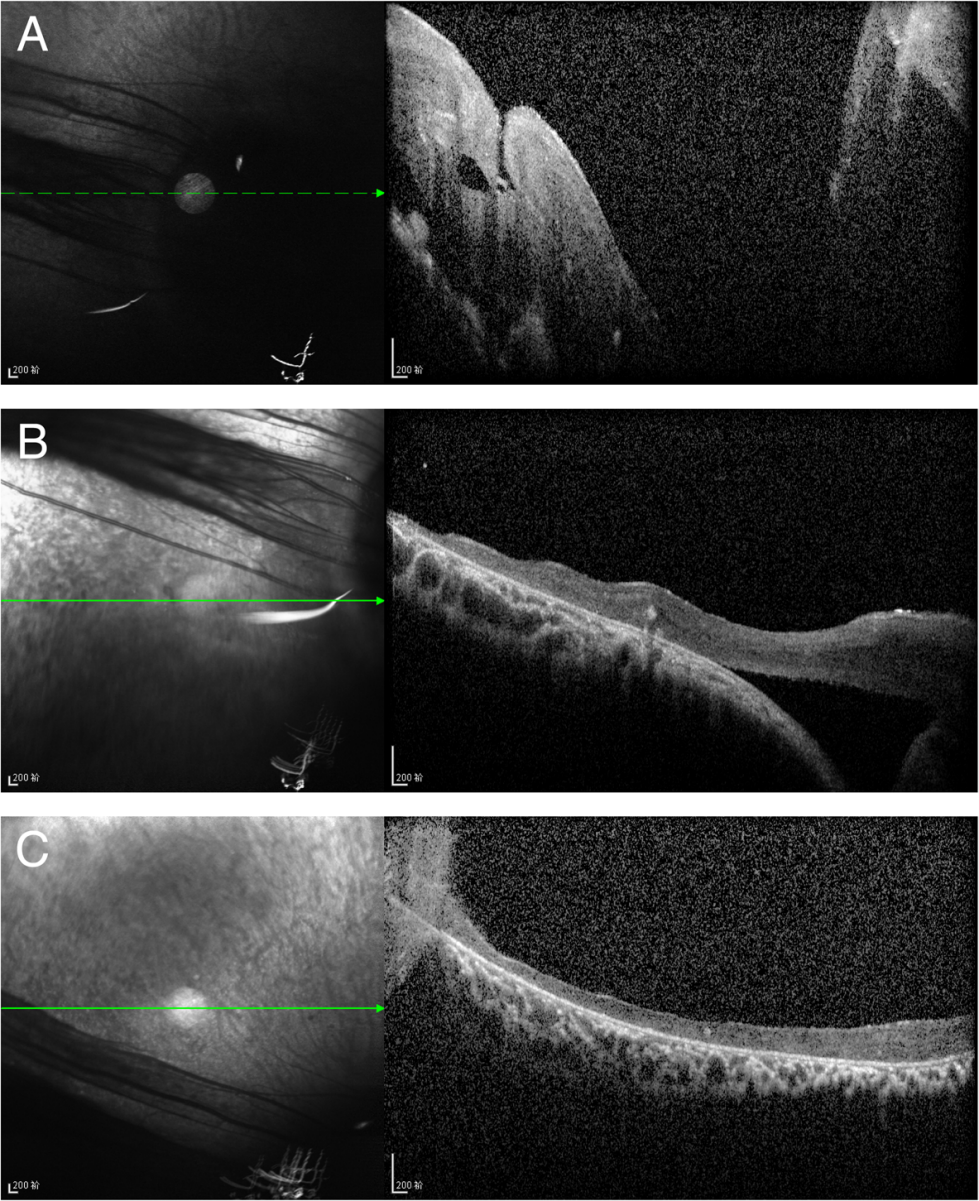
OCT of the RE. **a** A funnel-shaped excavation at the center of the optic disc. **b** A small amount of subretinal fluid at the margin of the optic disc. **c** Supratemporal peripapillary retina was reattached

On November 21, 2016, the patient returned 4 months post operation. Fundus fluorescein angiography (FFA) showed that the diameter of the right optic disc was about three times the normal size (Fig. 5a, b). The optic disc was hypofluorescent in the early phase. However, there was irregular late hyperfluorescence at the center and the edge of the optic disc and numerous vessels emanated from superotemporal and inferonasal margin of the disc (Fig. 5a, b). Optical coherence tomography (OCT) exhibited a funnel-shaped excavation at the center of the optic disc (Fig. 6a) with a small amount of preoperative subretinal fluid at the margin (Fig. 6b), and reattachment of peripheral retina (Fig. 6c).

When she visited us last November, 2 years after the surgery, the visual acuity was the same and there was no exacerbation of atrophy. OCT showed a bit of subretinal fluid in supratemporal parapapillary retina. There was no obvious retinal detachment in the rest of the retina and the retinal hole we made during the surgery was surrounded by laser spots. Her parents refused the second surgery considering that the right eye was stable. Beyond that, we observed some emulsified silicone oil in the anterior chamber, but not much.

## Discussion and conclusions

Here, we present a case of morning glory syndrome that is associated with PFV and retinal fold. Before surgery, vitreous opacities, retinal detachment, and temporal retinal folds were observed. A puzzling blurry fundus made it difficult to diagnose. However, according to B-ultrasound, the optic disc depression suggested the possibility of congenital optic nerve dysplasia. Moreover, temporal retinal folds also complicated the diagnosis. Importantly, MGS could be associated with several congenital ocular diseases, including PFV, congenital cataract, lid haemangioma, preretinal gliosis, lenticonus, and microphthalmia [9].

PFV may be fundamentally associated with MGS. Fei P et al. [9] reported that persistent fetal vasculature had a relatively high incidence compared to other congenital ocular anomalies in MGS patients. They also inferred that the regression of the hyaloid vasculature was easily compromised by the presence of optic disc defects which led to MGS. Furthermore, Cogan D et al. [10] hypothesized that the mass of white tissue on the abnormal morning glory disc was derived from the primitive hyaloid system. Based on the location of the vascular abnormalities, PFV could be divided into anterior, posterior, and combined PFV [11]. Anterior PFV is characterized by cataracts and retrolental opacity. Shallow anterior chamber and elongation of ciliary processes may occur in a few cases. Posterior PFV mainly involves the vitreous and the retina. It may manifest as a stalk from the optic nerve, retinal proliferative membrane, retinal fold, retinal detachment, or optic nerve hypoplasia. Macular abnormalities have also been reported [8]. The primary vitreous proliferates along the Cloquet’s canal and adheres to the retina, resulting in the formation of retina folds. This can cause partial retinal traction and thus leading to tractional retinal detachment [12]. Combined PFV involves both the anterior and posterior segments. Considering preoperative B-ultrasound scans and surgical findings, posterior PFV was more likely in this case. And retinal fold could be an indicator of posterior PFV. Despite the frequently occurring of PFV, retinal fold is not commonly seen in MGS.

Secondary RD occurs in about one-third of all MGS cases, however spontaneous resolution has also been observed [13]. Surgery is the preferred treatment for secondary RD. The glial tissue should be removed or trimmed as it causes traction on the retina within the defect and therefore raises the chance of subsequent RD [14]. The origin of the subretinal fluid is in dispute. Two hypotheses regarding this issue are that the fluid comes from cerebrospinal fluid [7, 15, 16] or from the vitreous cavity [17–19]. In addition, management of secondary RD is controversial. We chose to perform a retinotomy to remove the subretinal fluid, and results from follow-up visits were positive.

## Data Availability

All data and supplementary information are included in this published article.

## Abbreviations

BCVA: Best corrected visual acuity
FFA: Fundus fluorescein angiography
LE: Left eye
MGS: Morning glory syndrome
MRI: Magnetic resonance imaging
OCT: Optical coherence tomography
PFV: Persistent fetal vasculature
PPV: Pars plana vitrectomy
RD: Retinal detachment
RE: Right eye
